# Linking the relationship between drug-induced osteoporosis and the gut microbiota

**DOI:** 10.3389/fendo.2026.1818207

**Published:** 2026-06-01

**Authors:** Monika Martiniakova, Anna Sarocka, Noemi Penzes, Vladimira Mondockova, Aneta Sevcikova, Sona Ciernikova, Veronika Kovacova, Roman Biro, Joanna Folwarczna, Radoslav Omelka

**Affiliations:** 1 Department of Zoology and Anthropology, Faculty of Natural Sciences and Informatics, Constantine the Philosopher University in Nitra, Nitra, Slovakia; 2 Department of Botany and Genetics, Faculty of Natural Sciences and Informatics, Constantine the Philosopher University in Nitra, Nitra, Slovakia; 3 Department of Genetics, Cancer Research Institute, Biomedical Research Center of Slovak Academy of Sciences, Bratislava, Slovakia; 4 Department of Pharmacology, Faculty of Pharmaceutical Sciences in Sosnowiec, Medical University of Silesia in Katowice, Sosnowiec, Poland

**Keywords:** gut microbiota, osteoporosis treatment, pharmacological drugs, relationship, secondary osteoporosis

## Abstract

Drug-induced osteoporosis is considered secondary osteoporosis caused by pharmacological drugs that can alter the diversity and function of the gut microbiota (GM), which in turn may be associated with the development or exacerbation of osteoporosis. This review uncovers the relationship between drug-induced osteoporosis and GM based on preclinical and clinical studies. In this context, we focused on secondary osteoporosis induced by glucocorticoids, aromatase inhibitors, proton pump inhibitors, antiretroviral drugs, antiepileptic drugs, antipsychotics, antidepressants, and subsequent alterations in the GM. Different pharmacological drugs can induce secondary osteoporosis through multiple mechanisms, and some of them exert similar mechanisms of their harmful effect on bone health, including decreased osteoblastogenesis, increased osteoclastogenesis, disturbances in calcium and vitamin D metabolism, alterations in hormone and cytokine levels. In addition, diverse drugs can significantly reshape gut microbial communities, often in a drug- and context-specific manner. However, the mechanisms linking individual drugs, GM, and bone health are still largely unresolved. There is little or no direct evidence that drug-induced GM alterations mediate changes in bone turnover, bone mineral density (BMD), or fracture risk for most of the drug classes mentioned. Observational and interventional clinical studies in this area are necessary to provide conclusive evidence of the association between drug-induced osteoporosis and GM. Therapeutic approaches that have shown promise in the treatment of medication-induced osteoporosis include pharmacological interventions, adequate calcium and vitamin D intake, weight-bearing exercise, and preventive monitoring of BMD. Probiotic and prebiotic supplementation may be a future option if supported by compelling clinical evidence.

## 1 Introduction

Deterioration of bone microarchitecture and reduction in bone mass, manifested by decreased bone mineral density (BMD), are hallmarks of osteoporosis, a systemic metabolic skeletal disease that reduces bone strength and increases susceptibility to fractures. Osteoporotic fractures (also known as fragility fractures) mostly affect the wrist, hip, and vertebrae, and can cause great pain and suffering, as well as disability and occasionally death. As a result, osteoporosis represents a serious global health concern (1, 2). The worldwide prevalence of osteoporosis is approximately 18.3%, with demonstrable regional variations. More than two hundred million individuals are estimated to exhibit osteoporosis, which is related to a significant number of fractures each year, including 1.66 million hip fractures (3, 4). The underlying molecular mechanisms of osteoporosis are thought to result from either reduced activity of osteoblasts, elevated activity of osteoclasts, or both. This imbalance in the bone remodeling unit leads to impaired bone formation and accelerated bone resorption (5). A complex interaction of biomolecules, including cytokines such as receptor activator of nuclear factor kappa-B ligand (RANKL), osteoprotegerin (OPG), interleukins (ILs); growth factors including transforming growth factor-beta (TGF-β), insulin-like growth factor-1 (IGF-1); as well as changes in concentrations of hormones such as estrogen, testosterone, parathyroid hormone (PTH), mediates this disruption. The aforementioned molecules alter the local bone microenvironment and systemic bone metabolism in addition to regulating the growth, activity, and differentiation of osteoblasts and osteoclasts (3, 6).

Sex is an important factor when referring to osteoporosis. The two main forms of primary osteoporosis in women are type 1 (postmenopausal osteoporosis) and type 2 (senile osteoporosis). Estrogen deficiency causes postmenopausal osteoporosis, the most prevalent type of primary osteoporosis. Senile osteoporosis manifests after the age of 70 and its prevalence is 2:1 in women and men, respectively (7, 8). Men benefit from delayed bone loss because their levels of testosterone and estradiol decrease more slowly than those of women. Consequently, they experience fractures roughly ten years later than women. However, up to 40% of all osteoporotic fractures occur in men. Furthermore, men are twice as likely as women to face hip fracture-related mortality and morbidity (9). Secondary osteoporosis can result from secondary factors contributing to bone loss, including a variety of clinical and lifestyle issues. It has been reported that more than 50% of premenopausal women, 30% of postmenopausal women, and two-thirds of older men suffer from secondary osteoporosis (10, 11). Identifying secondary causes of osteoporosis is essential because treatment for such patients depends on the underlying conditions. In general, a number of conditions can contribute to secondary osteoporosis, such as endocrine, neuromuscular, gastrointestinal disorders, chronic inflammatory and nutritional conditions, chronic kidney disease, cancer, genetic conditions, medications, and adverse lifestyle factors. It is widely recognized that chronic use of various drugs (e.g., glucocorticoids, aromatase inhibitors, proton pump inhibitors, antiretrovirals, anticonvulsants, antipsychotics, antidepressants) can lead to reduced BMD, a main contributor to osteoporosis (11, 12).

**Figure 1:**
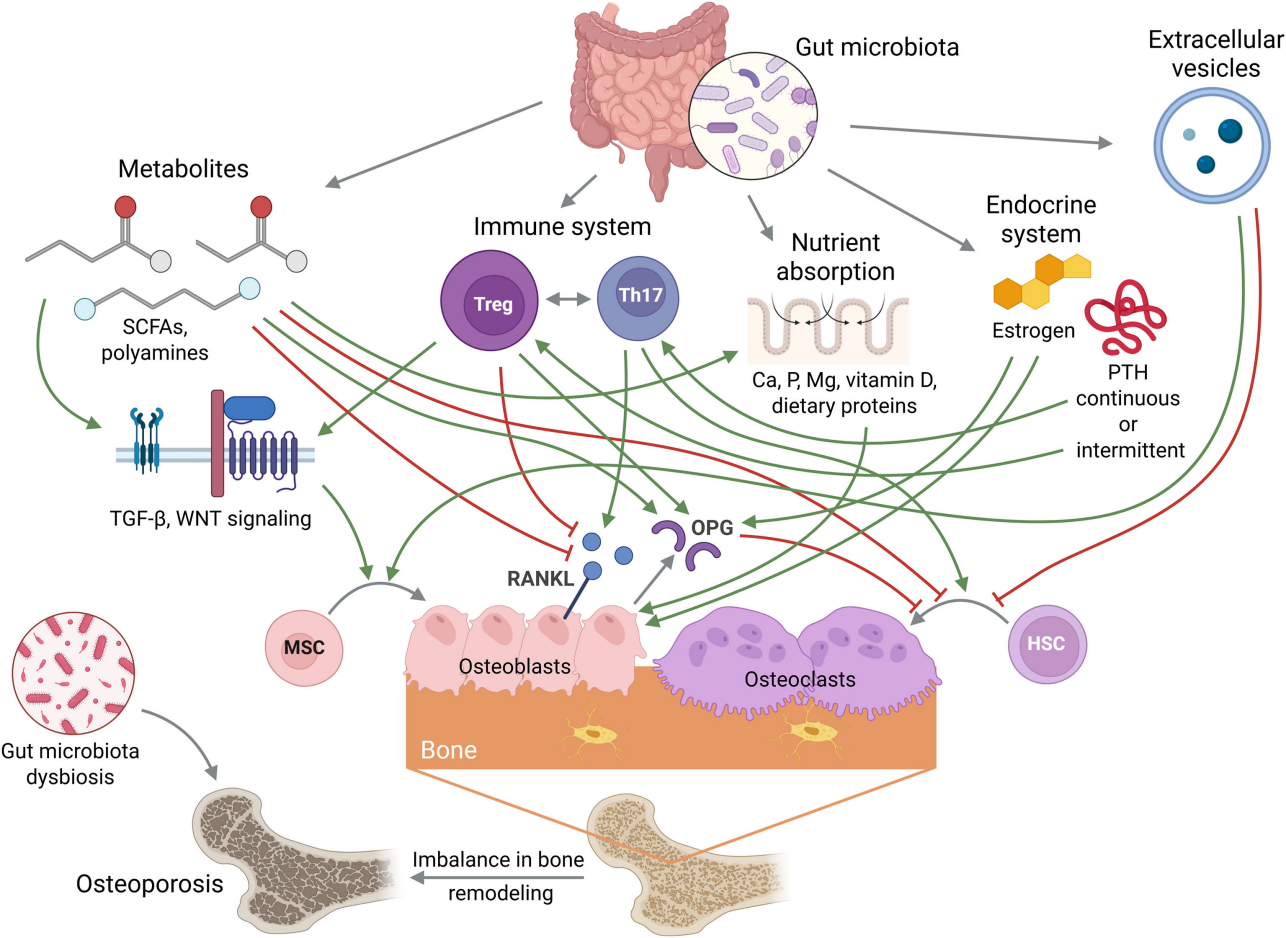
The gut-bone axis in the context of bone health and osteoporosis development. The GM regulates bone remodeling through microbial metabolites, interaction with immune cells, influencing nutrient absorption, regulation of hormonal activity (especially estrogen and PTH), and the release of extracellular vesicles. The metabolites produced (such as SCFAs or polyamines) suppress osteoclast number and differentiation and promote osteogenic differentiation by activating signaling pathways, including the TGF-β or Wnt pathways. In addition, SCFAs enhance nutrient absorption and act as signaling molecules that regulate osteoclastogenesis through inhibition of RANKL expression and promotion of OPG production. The GM also regulates bone metabolism by modulating the balance between Th17 and Treg immune cells. Th17 cells promote osteoclast differentiation by producing RANKL and pro-inflammatory cytokines, leading to increased bone resorption. Conversely, Treg cells inhibit osteoclastogenesis and elevate bone formation by secreting anti-inflammatory cytokines, TGF-β, enhancing the production of OPG, and downregulating the expression of RANKL. In addition, the GM supports the absorption of essential nutrients for skeletal development, including Ca, P, Mg, vitamin D, and dietary proteins. Regarding the regulation of hormonal activity, estrogen favors bone formation, suppresses RANKL in specific immune cells, and increases OPG release in osteoblasts. The GM can directly regulate host sex steroid levels by producing estrogen-metabolizing enzymes. On the other hand, the effect of PTH on bone remodeling depends on the route of exposure. Intermittent administration of PTH activates immune cell signaling, which in turn increases bone formation. Conversely, continuous administration of PTH has the opposite effect. In this context, some metabolites are essential for intermittent PTH delivery to stimulate bone formation; however, the GM can also amplify inflammatory responses by interacting with continuous PTH levels in the gut, leading to bone loss. The protective effect of several gut microbes on bone health may ultimately be mediated by the secretion of extracellular vesicles, which can accumulate in bone tissue and inhibit osteoclastogenesis and promote osteogenesis. Blunt red arrows indicate an inhibitory effect, sharp green arrows designate a stimulatory effect. Grey arrows indicate the connection of individual mechanisms. Ca, Calcium; GM, Gut microbiota; HSC, Hematopoietic stem cells; Mg, Magnesium; MSC, Mesenchymal stem cells; OPG, Osteoprotegerin; P, Phosphorus; PTH, Parathyroid hormone; RANKL, Receptor activator of nuclear factor kappa B ligand; SCFAs, Short-chain fatty acids; TGF-β, Transforming growth factor-beta; Th17, T helper cells 17; Treg, T Regulatory cells. Created with BioRender.com.

Growing data in recent years suggests that the gut microbiota (GM) plays a significant role in regulating bone homeostasis and that interactions between the skeletal system and GM may influence bone health. Current studies on the gut-bone axis (**Figure 1**) in the multifactorial etiology of osteoporosis have elucidated how the GM can affect bone metabolism through immune modulation, metabolic product formation, and nutrient absorption, thus revealing a potential pathway for regulating bone health (13, 14). Short-chain fatty acids (SCFAs) in the gut, such as butyrate and propionate, have been shown to directly contribute to bone remodeling by promoting osteoblast differentiation and proliferation, while inhibiting osteoclast formation (15, 16). Furthermore, by influencing the host immune system, specifically the balance between T helper 17 (Th17) cells and regulatory T (Treg) cells and the host inflammatory state, the GM indirectly influences the development of osteoporosis. Additionally, the GM regulates bone remodeling through its interactions with PTH and estrogen, which may indirectly cause osteoporosis (17–19). Although our understanding of the role of GM in osteoporosis has advanced significantly, there is still a lack of systematic knowledge about the precise mechanisms by which microbes regulate bone health, as well as the relationship between gut microbial diversity and specific bone pathologies. Numerous investigations have assessed the diversity and abundance of bacterial populations in the gut of patients with primary osteoporosis. Accordingly, these patients appear to have lower microbial diversity, with decreases in *Bacteroides* and *Roseburia* spp. and an increase in some species such as *Fusobacterium*, *Dialister*, *Faecalibacterium* and *Tolumonas* (20–22). However, studies examining the connection between drug-induced (secondary) osteoporosis and GM are quite limited. In this regard, it is known that drug-induced disruptions in the GM can alter metabolic profiles and bacterial diversity, possibly affecting gut permeability and metabolite release. Through the activation of immune cells, this series of events may influence bone mineralization and subsequently BMD as well (23).

Therefore, this review aimed to provide a comprehensive overview of the available information on the links between drug-induced osteoporosis and GM based on preclinical and clinical studies. In this context, we focused on secondary osteoporosis induced by glucocorticoids (GCs), aromatase inhibitors (AIs), proton pump inhibitors (PPIs), antiretroviral drugs (ARs), antiepileptic drugs (AEDs), antipsychotics (APs), antidepressants (ADs), and subsequent alterations in the GM. For greater comprehensiveness, this review also includes recommended therapy for drug-induced osteoporosis.

## 2 Glucocorticoid-induced osteoporosis and the gut microbiota

Glucocorticoids (GCs) are a group of endogenous hormones and/or immunomodulatory drugs, which primarily function through the glucocorticoid receptor to exert anti-inflammatory, anti-proliferative, and immunosuppressive properties (24). By reducing the activity of T cells and macrophages and inhibiting neutrophil adhesion, GCs suppress the transcription of genes linked to immune and inflammatory responses (12). Their adaptability in treating a wide range of acute and chronic inflammatory conditions, such as rheumatoid arthritis (RA), inflammatory bowel disease (IBD), alleviating the side effects of chemotherapy, and preventing rejection of transplanted organs, makes them a valuable therapeutic option (25). GCs can be classified by their duration of action into three groups: short-acting (cortisone, hydrocortisone), intermediate-acting (prednisone, prednisolone, methylprednisolone, triamcinolone, deflazacort), and long-acting (dexamethasone, betamethasone) (26). However, chronic GC therapy can lead to a number of negative effects, particularly glucocorticoid-induced osteoporosis (GIOP), which is the most prevalent type of secondary osteoporosis (27).

Depending on the dosage, duration of use, and underlying medical condition being treated, GCs may have cumulative adverse effects on bone health predominantly through the suppression of the Wnt signaling pathway, modulation of the RANKL/OPG signaling pathway, stimulation of macrophage colony-stimulating factor (M-CSF) production, and apoptosis of osteoblasts and osteocytes (12, 24). Suppression of the Wnt pathway can lead to inhibition of osteoblastogenesis and stimulation of adipogenesis through peroxisome proliferator-activated receptor gamma (PPARγ) activation (28, 29). The increase in osteoclast numbers is facilitated by elevated expression of RANKL, stimulated by granulocyte colony-stimulating factor (G-CSF), and the simultaneous suppression of OPG (30). In addition, GCs exert a direct anti-apoptotic effect on mature osteoclasts and increase the production of M-CSF, which is a key protein for osteoclast survival and activity (12). GCs also influence osteocyte viability and function, thereby reducing bone resistance and delaying bone repair (31). Furthermore, GC treatment can lead to a decrease in calcium (Ca) levels due to reduced intestinal Ca absorption and increased urinary Ca excretion, which may result in mild secondary hyperparathyroidism, reduced levels of IGF-1, growth hormone, estrogen, and elevated osteoclast activity (12, 32). Pharmacological dosages of GCs may also raise the intracellular oxidative stress milieu (33–35).

Numerous studies have demonstrated that GCs are consistent with a considerable decrease in BMD with a rapid rise in vertebral and non-vertebral fractures during treatment, even at low doses. According to Cooper (36), continuous GC therapy (over 90% of prednisolone) increased the risk of fractures and decreased BMD. Furthermore, the risk of vertebral fractures has been observed even with very low doses of prednisolone (< 2.5 mg/day). A meta-analysis by Lems et al. (37) revealed that GC-treated patients with chronic inflammatory diseases (including RA, IBD, systemic lupus erythematosus, polymyalgia rheumatica, vasculitis, granulomatosis with polyangiitis) experienced a higher risk of osteoporotic fractures. The mean bone loss during a one-year period was -1.7% in the lumbar spine and -1.3% in the femoral neck. In RA patients treated with GCs, a significant reduction in BMD of the lumbar spine and femoral neck was found compared to the RA control group. Furthermore, these patients had a prevalence of vertebral fractures of 13% (38). GC therapy in subjects with IBD has been associated with an elevated risk of vertebral fractures (39) as well as hip fractures (40). According to Mori et al. (41), individuals receiving GCs for rheumatological conditions may be at risk for vertebral fractures due to long-term disease duration, low body mass index (BMI), and reduced total hip BMD. In a study by Amiche et al. (42), patients who started taking GCs had a higher likelihood of vertebral fractures and decreased BMD compared with chronic GC users, indicating that fracture incidence among GC users may be more common than previously estimated. Indeed, GC initiators experience a rapid phase of bone loss in the first year (a 6-12% decrease in lumbar spine BMD), followed by a slower but continuous decline (24). The risk of fracture is rising early after initiation of GC therapy (within three to six months) and declines soon after its stopping (43, 44).

In general, GCs are known to alter the composition of the GM, leading to dysbiosis (45, 46). Zhang et al. (47) analyzed how long-term prednisone treatment alters the GM and microbial metabolites in a rat model. After six weeks of prednisone treatment, the genus *Anaerobacterium* was increased, while *Eisenbergiella, Alistipes*, and *Clostridium XIVb* were reduced. Additionally, fecal SCFAs were decreased in GC-treated animals. Prednisolone treatment also altered GM composition in a murine model, reducing *Verrucomicrobiales* and *Bacteroidales* while increasing *Clostridiales* (46). Chen et al. (48) reported that methylprednisolone administration reduced both the absolute and relative abundance of the genus *Lactobacillus*. Armir et al. (49) proposed an *in silico* mining tool to predict drug metabolism mediated by xenobiotic-metabolizing enzymes encoded across bacterial species in the Unified Human Gastrointestinal Genome (UHGG) dataset. Their analysis suggested that deflazacort may be metabolized by members of the phyla *Bacteroidota, Verrucomicrobiota, Proteobacteria, Firmicutes_C, Firmicutes*, and *Firmicutes_A*. In dystrophic mice, deflazacort administration increased the abundance of *Desulfovibrionaceae* while decreasing *Erysipelotrichaceae* and *Burkholderiaceae* (50). Dexamethasone administration has been shown to alter gut microbial community structure over four weeks, increasing *Bifidobacterium* and *Lactobacillus* while eliminating the mucin-degrading genus *Mucispirillum* (51).

Although the GM influences and regulates BMD, knowledge about the cross-linking relationship with GIOP remains limited. Many patients on long-term GC therapy never receive preventive treatment for potential bone loss, and some only begin therapy after experiencing a fracture (52). Therefore, gut microbes might represent a novel target for mitigating the side effects associated with high-dose GC therapy (53). Preclinical studies have demonstrated that the modulation of the gut-bone axis might prevent GIOP (54). Interestingly, the effect of Korean red ginseng extract was evaluated in a murine model of GIOP. One month of GC therapy led to trabecular bone loss, damaged intestinal barrier, and altered immune cell populations. The bacterial species *Turicibacter sanguinis* was detected only in GC-treated mice, whereas treatment with Korean red ginseng prevented bone loss and GIOP *in vivo*. Sequencing of fecal samples demonstrated enrichment of *Lactobacillus johnsonii* in the treated animals (54). It was found that administration of *Lactobacillus reuteri* prevented prednisolone-induced trabecular bone loss *in vivo*. Similarly, the oral administration of *Lactobacillus plantarum* LP45 improved femoral biomechanics in rats with GIOP (55). However, a clinical trial in healthy young adults treated with prednisolone found that *Lactobacillus reuteri* supplementation did not significantly affect bone turnover markers, including osteocalcin (OCN), C-terminal telopeptide of type I collagen (CTx), and procollagen type 1 N-terminal propeptide (P1NP), compared to placebo (56). Notably, a six-week supplementation with *Lactobacillus animalis* mitigated methylprednisolone-induced osteonecrosis of the femoral head by enhancing osteogenesis (48). Protective effects of Shuanghe Decoction (SHD), a traditional Chinese medicinal formulation, were observed in a rat model of methylprednisolone-induced osteonecrosis of the femoral head. Administration of SHD enhanced bone morphology and elevated the expression of osteogenic markers, including runt-related transcription factor 2 (RUNX2), OCN, and collagen type I alpha 1 (COL1A1). In parallel, SHD modulated the gut microbial community, notably influencing the relative abundances of *Verrucomicrobia, Allobaculum*, and taxa within *Burkholderiales* (57). The effects of *Lactobacillus plantarum* on bone health have been investigated in an animal model of dexamethasone-induced osteoporosis, showing that probiotic administration reshapes the GM by increasing beneficial taxa such as *Ruminococcus, Lachnospiraceae_NK4A136_group, UCG_005, Romboutsia*, and *Christensenellaceae_R-7_group*, while reducing *Desulfovibrionaceae*. Probiotic supplementation resulted in higher BMD and trabecular number. In addition, serum concentrations of metabolites that promote osteoblastogenesis and suppress osteoclastogenesis, including pyrazine and γ-glutamylcysteine, were elevated. These findings indicate that *Lactobacillus plantarum* mitigates dexamethasone-induced osteoporosis by modulating the gut-bone axis, enhancing beneficial microbes, reducing harmful taxa, and increasing levels of bone-protective metabolites (58).

## 3 Aromatase inhibitor-induced osteoporosis and the gut microbiota

Aromatase inhibitors (AIs) are commonly prescribed for the management of estrogen receptor-positive breast cancer in postmenopausal women. Current evidence has demonstrated their efficacy when employed as adjuvant therapy in combination with chemotherapy and surgical interventions for the treatment of metastatic, estrogen receptor-dependent breast cancer (59). In general, AIs lower circulating estrogen levels by suppressing the activity of the aromatase enzyme, a member of the cytochrome P450 superfamily (CYP19A1), which catalyzes the peripheral conversion of androgens to estrogens (64). Reversible non-steroidal AIs (letrozole, anastrozole) and steroidal irreversible AIs (exemestane) are clinically used AIs (60, 61). Prolonged AI therapy is linked to adverse side effects, such as a number of autoimmune disorders, and musculoskeletal system damage, which negatively affect bone health through accelerated bone loss and thus raise the risk of osteopenia, osteoporosis, and bone fractures (62, 63).

Estrogen deficiency caused by AI treatment disrupts bone remodeling mechanisms (64, 65). Generally, estrogen inhibits RANKL-induced osteoclast differentiation, prevents osteoblast apoptosis, and supports the maturation and differentiation of osteoblast precursor cells (66, 67). Additionally, estrogen suppresses the production of IL-1 and tumor necrosis factor-alpha (TNF-α) (12). A decrease in estrogen levels, therefore, leads to elevated bone resorption and subsequently osteoporosis, especially in the trabecular bone compartments (2).

A meta-analysis by Lee (68) found that AI therapy in patients with breast cancer significantly increased the risk of osteoporotic fractures, with the most pronounced risk noted for vertebral fractures. In non-osteoporotic postmenopausal women with breast cancer, long-term AI treatment considerably reduced BMD, lumbar spine trabecular bone score (TBS), and hip geometry (69). Postmenopausal women with hormone receptor-positive early breast cancer who received letrozole for about five years experienced a greater incidence of bone pain, bone fractures, and new-onset osteoporosis, according to Goss et al. (70). In a study by Servitja et al. (71), postmenopausal women with early breast cancer treated with AIs (letrozole 68.8%, exemestane 30.1%, and anastrozole 1.1%) exhibited unrecognized vitamin D deficiency. Furthermore, 53.7% of women had osteopenia, and 19.4% had osteoporosis. The aforementioned findings highlight the need for routine measurement of 25-hydroxyvitamin D levels in these patients and administration of supplements when necessary. Increased markers of bone resorption (urinary pyridinoline – PYD and urinary deoxypyridinoline - DPD) and decreased PTH were observed in postmenopausal women with breast cancer treated with letrozole (72). It has been shown that various types of AIs may have different impacts on bone health. After two years of treatment with anastrozole (1 mg/day) or exemestane (25 mg/day), anastrozole had a more detrimental effect on BMD of the lumbar spine and hip (73). On the other hand, anastrozole (1 mg/day) had a less deleterious impact on clinical fractures compared with letrozole (2.5 mg/day) over 5 years of therapy (74). When comparing all three drugs over 5 years, the use of anastrozole (1 mg/day) had a smaller effect on the development of osteoporosis compared to letrozole (2.5 mg/day) and exemestane (25 mg/day). However, none of the three AIs was superior to the others in terms of effectiveness (75).

Treatment with AIs has been associated with alterations in gut bacterial diversity and changes in taxa involved in estrogen metabolism (76). In breast cancer patients who underwent surgery and radiation, oral AI administration did not significantly affect α- or β-diversity. However, Cook et al. (77) documented enrichment of *Parabacteroides merdae*, *Ruthenibacterium lactatiformans*, and *Oscillospiraceae* after AI therapy, while the beneficial butyrate-producing bacterium *Faecalibacterium prausnitzii* was significantly decreased. Gut dysbiosis in breast cancer patients may be exacerbated by AI therapy (78). Lasagna et al. (79) studied the association between GM and inflammation in postmenopausal women with breast cancer receiving AI treatment. Elevated levels of *Veillonella* were found in treated patients compared to healthy controls. Comparison of gut bacterial composition between AI-treated postmenopausal breast cancer patients (receiving anastrozole, letrozole, or exemestane) and healthy controls showed significant enrichment of *Bifidobacterium animalis* in patients, whereas taxa such as *Coprococcus, Ruminococcus, Corynebacterium, Butyricicoccus, Holdemania*, and *Haemophilus* were present at lower levels (80). In a rat model, selenium nanoparticles conjugated with anastrozole reduced bone damage and decreased osteoblast death (81). The specific relationship between GM alterations and AI-induced osteoporosis remains to be elucidated. Maintaining a well-balanced GM enriched with beneficial bacteria, or modulating the GM toward a more favorable composition, may help support bone health in breast cancer survivors (82). Recently, an ongoing clinical trial (NCT07044310) is evaluating whether probiotic supplementation can prevent bone loss in 38 participants with early-stage hormone receptor-positive breast cancer initiating AI therapy.

## 4 Proton pump inhibitor-induced osteoporosis and the gut microbiota

Proton pump inhibitors (PPIs), including omeprazole, esomeprazole, lansoprazole, and pantoprazole, belong to the most widely prescribed drugs for managing gastrointestinal disorders, such as dyspepsia, gastroesophageal disease, gastric ulcers, and *Helicobacter pylori* infection (83, 84). They are also utilized to prevent gastric injuries caused by non-steroidal anti-inflammatory drugs and surgical procedures, primarily by reducing gastric acid release through inhibition of the hydrogen/potassium adenosine triphosphatase enzyme system (H^+^/K^+^-ATPase) in gastric cells (85, 86). Current evidence suggests that long-term PPI usage may rise the incidence of osteoporotic fractures and alter bone metabolism by blocking the absorption of vitamins and trace elements in the digestive system (85, 87).

Several potential mechanisms have been identified by which PPIs can interfere with bone health, including impaired intestinal absorption of Ca and magnesium (Mg), disruption of osteoclast function via inhibition of osteoclast vacuolar-type-ATPase, and subsequently declined BMD (88). Prolonged use of PPIs was associated with negative Ca balance due to increased gastric pH, resulting in elevated bone resorption and lower BMD (85). Hypomagnesemia can disrupt the PTH-vitamin D axis, which negatively impacts bone health by interfering with bone remodeling and Ca homeostasis (88, 89). In addition, PPI-induced vitamin B12 deficiency has also been reported, leading to elevated homocysteine ​​(HCY) levels and disturbed collagen cross-linking, making bones more fragile and susceptible to fractures (90). Additionally, prolonged gastric acid suppression may increase PTH levels, which stimulate bone resorption (96–98). Inhibition of vacuolar-type-ATPase in osteoclasts due to PPIs use eliminates the secretion of hydrogen (H+) by osteoclasts, thereby reducing their ability to resorb bone (91, 92).

According to the US Food and Drug Administration (FDA), a potentially elevated risk of hip, wrist, and spine fractures has been reported with long-term use of PPIs (93). Fattahi et al. (94) found that patients (healthy men and women, with more than 80% of patients being women) who used PPIs for more than two years had lower BMD in the femoral neck. In a study by Gray et al. (95), PPI therapy in postmenopausal women was not linked to hip fractures but was modestly associated with spine, wrist, and total fractures. An increased incidence of vertebral fractures has been observed in postmenopausal women taking omeprazole (96). In contrast, Hansen et al. (97) reported that prolonged PPI treatment (dexlansoprazole - 60 mg and esomeprazole - 40 mg for 26 weeks) increased markers of bone formation and bone resorption, including P1NP and CTx, but did not affect BMD (in the lumbar spine, femoral neck, and total hip) in healthy postmenopausal women. An umbrella review by Alanazi et al. (98) revealed that PPI users (menopausal and postmenopausal women, children and young adults, hemodialysis patients, patients undergoing dental implants) are more likely to have wrist, hip, and spine fractures. A longer PPI prescription and higher cumulative PPI dose were also linked to elevated fracture risk (including almost all fracture types) in patients with chronic kidney disease younger than 60 years (99). Bioletto et al. (100) point to a sex-specific association of chronic PPI use with lower BMD and deteriorated bone quality. In men, PPI therapy was associated with poorer trabecular bone quality even after adjusting for BMD at the lumbar spine and femoral neck. However, no association was observed in women.

The impact of long-term PPI use on the GM may be mediated by alterations such as hypomagnesemia and hypocalcemia (87). Reduced gastric acidity caused by PPI treatment can increase susceptibility to gastrointestinal infections, including *Clostridium difficile* infection (101). Additional negative effects of PPIs may result from changes in the gastric microbiome, small intestinal bacterial overgrowth (SIBO), and impaired immune responses (102). PPI use has been associated with reduced gut bacterial diversity and enrichment of taxa including *Enterococcus*, *Streptococcus*, *Staphylococcus*, and *Escherichia coli* (103). Furthermore, higher abundances of *Rothia* and *Streptococcus* have been documented in PPI users (104). A meta-analysis by Zhang et al. (105) confirmed that PPIs reduce gut microbial diversity and deplete members of the *Ruminococcaceae* and *Lachnospiraceae* families, which are key producers of SCFAs (106). Zhang et al. (107) investigated whether PPI use could influence BMD via alterations in gut microbes or metabolites. Several taxa, including *Streptococcus salivarius*, *Streptococcus parasanguinis*, *Rothia mucilaginosa*, and *Eubacterium* sp. OM08-24, were positively correlated with PPI use. PPI treatment was associated with reduced lumbar spine and total hip BMD; however, these changes were not mediated by the GM. Thus, while PPI use is linked to alterations in the GM, these microbial changes do not appear to directly influence bone parameters.

## 5 Osteoporosis induced by antiretroviral drugs and the gut microbiota

Antiretroviral therapy (ART) is used in the treatment of human immunodeficiency virus (HIV) and is highly effective in inhibiting HIV replication in patients who have access to and adhere to antiretroviral drugs (ARs). In general, ART usually includes non-nucleoside reverse transcriptase inhibitors (NNRTIs), nucleoside reverse transcriptase inhibitors (NRTIs), and protease inhibitors (PIs) (12, 108). Highly active ART (HAART) consists of a combination of more than three ARs. Combining two NRTIs (often tenofovir-emtricitabine) with one NNRTI or integrase strand transfer inhibitor (dolutegravir, raltegravir) is the standard of care for most previously untreated individuals (109). HAART can lead to sustained (and sometimes lifelong) suppression of viral replication (110). Despite the benefits and improved longevity, osteopenia and osteoporosis are among the most prevalent comorbidities in HIV patients (111). Reduced BMD, increased bone turnover, and both vertebral and non-vertebral fractures are often determined in individuals during the initial years of treatment (106, 112).

Adverse effects of ARs on bone health are mediated by multiple mechanisms. NNRTIs, including efavirenz and rilpivirine, are able to enhance the metabolism of 25-OH vitamin D through CYP450, leading to hypovitaminosis D. This can be associated with falls and elevated bone catabolism (113). By blocking DNA polymerase-γ, NRTIs (abacavir–lamivudine) cause hyperleptinemia and the accompanying mobilization of calcium hydroxyapatite, which results in chronic acidosis (114). Additionally, NRTIs also influence osteoclasts by upregulating RANKL, which contributes to bone loss (12). PIs, including ritonavir, darunavir, and lopinavir, are associated with reduced BMD and bone loss (115). Furthermore, PIs increase the lifetime of osteoclasts and decrease the number of osteoblasts via stimulating nuclear factor kappa B (NF-κB) (12). In a study by Vlot et al. (116), men with HIV infection who received extended HAART (emtricitabine, tenofovir, efavirenz, lopinavir, and ritonavir) had increased bone turnover and reduced BMD at the femoral neck and total hip. BMD loss was also observed in HIV patients who used two different ART (abacavir–lamivudine and tenofovir–emtricitabine) for 96 weeks. In addition, individuals treated with tenofovir-emtricitabine experienced greater decreases in BMD compared with those treated with abacavir-lamivudine (117). Komatsu et al. (118) reported that long-term tenofovir disoproxil fumarate users, including HIV-infected younger males and older postmenopausal women, had a higher incidence of osteoporosis-related fractures. HIV patients who received PIs were more likely to have reduced BMD and osteoporosis versus untreated individuals (119). In HIV-infected men, duration of PI (ritonavir) therapy was significantly correlated with lumbar spine BMD loss, while patients who discontinued PI therapy had significantly higher BMD (120). The significance of monitoring BMD during lifelong ART was highlighted by the fact that HIV-infected naive patients showed a higher reduction in lumbar spine BMD following a year of therapy with PIs as opposed to NNRTIs (121). Interestingly, switch to dolutegravir with rilpivirine was associated with significant improvements in lumbar spine and total hip BMD, as well as bone turnover markers, compared with a tenofovir-based triple combination in HIV-infected adults (122). Similarly, switching from tenofovir to raltegravir increased the BMD of the lumbar spine and total hip and reduced bone turnover markers in men with HIV (123). Additionally, switching from tenofovir to abacavir led to a slight improvement in femoral BMD in HIV patients (124). Changes in ART, along with Ca and vitamin D supplementation, lifestyle modification, and pharmacological treatment of osteoporosis (especially bisphosphonates), can therefore be used to mitigate bone loss in HIV patients (125).

ART treatment shifts GM composition in HIV-infected individuals (126, 127). Analysis of rectal swabs in HIV patients revealed a decrease in *Prevotella*, whereas genera such as *Peptoniphilus*, *Finegoldia*, and *Anaerococcus* were elevated (128). Pinto-Cardoso et al. (129) showed that individuals on long-term two ART regimens (NNRTIs or PIs combined with the same backbone of NRTIs) had reduced gut bacterial diversity. Notably, the beneficial bacterium *Faecalibacterium prausnitzii* from the family *Ruminococcaceae* was absent in ART-treated HIV patients compared to uninfected controls (129). *In vitro* studies indicate that zidovudine, an NRTI, exhibits antibacterial activity, suggesting a potential direct impact of ART on the GM (130). Importantly, HIV-associated dysbiosis has also been proposed as a contributor to bone disease (131). However, the extent to which modulation of gut dysbiosis in ART-treated individuals affects Ca homeostasis and mitigates bone loss remains unclear (132). Mei et al. (125) investigated whether specific GM compositions and their metabolites are associated with BMD in women receiving ART. Women with low BMD had higher levels of five bacterial genera, including *Dorea*, unclassified *Lachnospiraceae*, *Megasphaera*, *Mitsuokella*, and *Ruminococcus*, than those with normal BMD. Additionally, higher levels of several BMD-related metabolites (creatinine, retinol, N1-methyl-2-pyridone-5-carboxamide, dimethylglycine, 4-pyridoxic acid) were observed in patients with HIV infection, whereas metabolites such as homoarginine and serine were increased in uninfected individuals. An inverse correlation between *Megasphaera* abundance and dimethylglycine levels was also noted in women with HIV.

## 6 Osteoporosis induced by antiepileptic drugs and the gut microbiota

Antiepileptic drugs (AEDs), also known as anticonvulsants, are pharmacological agents that inhibit seizure activity and are therefore widely prescribed for the treatment of epilepsy (133). These medications modulate neuronal activity primarily by regulating ion channels and neurotransmitter systems, thereby stabilizing neuronal excitability. Seizures can be triggered by a variety of factors, including trauma, infection, stroke, and genetic mutations (134). Generally, AEDs can be divided into three categories based on their mechanism of action: drugs that increase inhibitory transmission by enhancing the action of gamma-aminobutyric acid (GABA) (phenobarbital, gabapentin, valproate), drugs that reduce excitatory neurotransmission by inhibiting the action of glutamate (Glu) (felbamate, perampanel, topiramate), and drugs that functions by stabilizing neuronal membranes through modulation of voltage-gated ion channels (phenytoin, carbamazepine, zonisamide, retigabine) (134–136). Anticonvulsants can also be classified based on their ability to induce liver enzyme CYP450 into two groups: enzyme-inducing AEDs (EI-AEDs, e.g., phenytoin, phenobarbital, carbamazepine) and non-enzyme-inducing AEDs (NEI-AEDs, e.g., valproate, gabapentin, levetiracetam) (133, 137). According to several studies, 11-31% of epileptic patients develop osteoporosis, and their abnormal bone metabolism increases the risk of fractures two- to six-fold. In this context, patients with epilepsy who use EI-AEDs are more likely to experience osteoporotic fractures. Although NEI-AEDs are linked to a higher incidence of fractures as well, the findings across studies are less consistent (138).

Overall, AEDs may contribute to secondary osteoporosis through multiple mechanisms: activation of pregnane X receptor (PXR), hypocalcemia, estrogen deficiency, and increased HCY levels (12). Activation of PXR by EI-AEDs stimulates CYP450 enzymes, such as CYP3A4, leading to reduced levels of active vitamin D, which can contribute to hypocalcemia (138). Low Ca levels trigger secondary hyperparathyroidism, which induces bone resorption by osteoclasts (139). Estrogen deficiency caused by AEDs can raise the production of pro-inflammatory cytokines (IL-1, IL-6, TNF-α), which subsequently trigger the release of RANKL and initiate osteoclastogenesis (140). Elevated HCY levels may disrupt collagen cross-linking and promote osteoclastogenesis (141). Several *in vitro* studies suggest that EI-AEDs (carbamazepine, phenytoin) can also directly inhibit osteoblastogenesis (141, 142). On the other hand, NEI-AEDs (valproate) may have the ability to activate osteoclasts, leading to bone loss (138, 143).

A high prevalence of vertebral fractures has been determined in male veterans with epilepsy chronically treated with AEDs (specifically phenobarbital, phenytoin, carbamazepine, primidone, or valproate) (144). According to Chandrasekaran et al. (145), men with epilepsy receiving various AEDs (phenytoin, carbamazepine, valproate, pregabalin, or clonazepam) had 9.1% lower adjusted mean BMD at the hip and lumbar spine compared to non-users. In women, BMD tended to be lower only at the hip. Conversely, women are more likely than men to experience negative effects on bone metabolism from EI-AEDs, such as lower levels of Ca, vitamin D, and PTH (146). Shi et al. (147) found that children undergoing AED monotherapy exhibited decreased markers of bone metabolism versus untreated controls. According to Simm et al. (148), children treated with AEDs had reduced tibial BMD and more fractures than their matched control group. An increased risk of hip fractures has been reported in patients (both epileptic women and men of various ages) receiving EI-AEDs (149). Griepp et al. (150) pointed out that EI-AEDs were more detrimental to bone health, as reflected by lower BMD at the femoral neck or lumbar spine compared to NEI-AEDs. However, Vera et al. (151) state that NEI-AED treatment with valproate may lead to low bone mass in pediatric patients, and adequate Ca intake may counteract such detrimental effects.

In patients with epilepsy, the relative abundances of *Proteobacteria*, *Verrucomicrobia*, *Fusobacteria*, and *Firmicutes* were elevated, whereas *Actinobacteria* and *Bacteroidetes* were reduced (152). Some AEDs possess antimicrobial properties, which can alter GM composition and bacterial ratios (153). Furthermore, both zonisamide and clonazepam are metabolized by gut microbes (154). Gong et al. (155) reported that three months of valproate administration increased the ratio of *Firmicutes* to *Bacteroidetes*. In naïve mice, topiramate treatment increased the abundance of the probiotic bacterium *Lactobacillus johnsonii*. Co-treatment with this probiotic and topiramate reduced pentylenetetrazol-induced seizures and elevated levels of butyrate, as well as members of the *Lachnospiraceae* family, key butyrate producers (156). In a soil collembolan (*Folsomia candida*) model, carbamazepine altered GM composition and increased the abundance of *Arthrobacter*, *Achromobacter*, *Gordonia*, and *Shinella*, which are linked to xenobiotic metabolism, thereby increasing the dissemination of antibiotic resistance genes (157). In the case of AED, there is no direct evidence that changes in GM may be associated with alterations in bone structure.

## 7 Osteoporosis induced by antipsychotic drugs and the gut microbiota

Antipsychotic drugs (APs) are primarily used to treat psychiatric disorders, particularly schizophrenia. Additionally, they are employed in the management of bipolar disorder, anxiety, borderline personality disorder, insomnia, attention deficit hyperactivity disorder, and substance use disorders (158–160). In general, APs are primarily categorized based on their affinity and interaction with dopamine and serotonin (5-hydroxytryptamine, 5-HT) receptors, although they also exhibit affinity for adrenergic, histamine, and muscarinic receptors (161). The classification includes first-generation antipsychotics (FGAs), second-generation antipsychotics (SGAs), and third-generation antipsychotics (TGAs). FGAs (chlorpromazine, haloperidol, trifluperazine, thioridazine, and fluphenazine) act as dopamine D2 receptor antagonists and are effective in treating positive symptoms (e.g., hallucinations, delusions (161, 162). SGAs (clozapine, quetiapine, olanzapine, risperidone) are D2 and 5-HT receptor antagonists and are more effective in reducing negative symptoms (e.g., anhedonia, social withdrawal) (163). TGAs (aripiprazole, ropinirole, brexpiprazole) function as partial agonists of D2, D3, and 5-HT1A receptors, rather than as antagonists, while simultaneously antagonizing 5-HT2A and 5-HT2B receptors (161). According to available information, more than 60% of patients receiving APs suffer from bone loss even in younger populations, increasing the risk of osteoporosis, particularly with long-term use (164, 165).

The precise mechanisms through which APs may impair bone health predominantly involve AP-induced hyperprolactinemia and other endocrine effects. In general, APs block dopamine receptors, leading to elevated prolactin levels, which may suppress estrogen (166). According to Wada et al. (167), approximately one-third of pediatric and adolescent patients with psychiatric disorders treated with APs exhibited abnormal prolactin levels. In prolactinoma patients, decreased BMD in the femoral neck and lumbar spine has been found (168). Raffin et al. (169) demonstrated that among adolescent psychiatric patients receiving SGAs, up to 60% had hyperprolactinemia and 89% experienced vitamin D deficiency. Furthermore, APs act on a variety of receptors, including dopamine D2, 5-HT, and adrenergic receptors, which are found predominantly in the brain but have also been found to be present in osteoblasts and osteoclasts (170). APs can interact with these receptors, potentially disrupting the balance between bone formation and bone resorption through modulation of multiple signaling pathways, as well as by affecting gonadotropins, leading to decreased BMD and increased fracture risk. APs can also induce sedation, resulting in reduced physical activity and subsequent muscle weakening (161, 171).

A narrative review by Mercurio et al. (164) showed that AP therapy reduced femoral neck and lumbar spine BMD, increased bone fragility, and raised vertebral and non-vertebral fracture risk. Similarly, Al-Omran et al. (165) revealed that patients of both sexes receiving APs had low bone mass, as reflected by decreased lumbar spine BMD. According to Lee and colleagues (172), use of any AP was associated with a nearly 1.5-fold elevated fracture risk regardless of study design, fracture site, or age group. Moreover, FGA users had a higher risk of hip fracture than SGA users, especially among elderly patients, which is also consistent with the results of Guo et al. (173). In contrast, long-term FGA users were not found to experience an increased risk of hip/femur fractures and non-hip/femur fractures compared with SGA users (174). According to Oderda et al. (175), prolonged treatment with both FGAs and SGAs was linked to an increased incidence of hip fractures, predominantly in older adults. Similarly, a meta-analysis of Papola et al. (176) found a 57% increase in the risk of hip fractures and a 17% increase in the risk of any fractures in individuals exposed to both FAPs and SGAs. In addition to an elevated risk of osteoporotic fractures, AP usage was linked to a higher risk of falls. Guo et al. (173) reported that SGAs were the class of drugs with the highest risk of falls in patients receiving them.

Manchia et al. (177) characterized the GM composition of patients with schizophrenia and healthy controls and evaluated the effects of FGAs and SGAs on GM in schizophrenia patients. The composition of the GM has been shown to be different in patients with schizophrenia and control subjects, with the abundance of bacteria at different taxonomic levels being exclusively in one group. Specific bacterial families, including *Paenibacillaceae*, *Cytophagaceae*, and *Morganellaceae*, were depleted in schizophrenia patients. At lower taxonomic levels, genera such as *Acetanaerobacterium*, *Haemophilus*, *Turicibacter*, *Obesumbacterium*, *Gracilibacter*, *Intestinibacter*, *Hespellia*, and *Weissella* were found exclusively in healthy controls. Similar results were also noted depending on treatment response and exposure to different classes of APs. Specifically, the FGA group had increased numbers of butyrate-producing bacteria, including *Erysipelotrichaceae*, *Butyricimonas*, *Blautia*, and *Paraprevotella*, compared to the SGA group. However, the GM of the latter group was strongly enriched in another butyrate producer, *Faecalibacterium prausnitzii*, with potential anti-inflammatory effects. A systematic review by Skonieczna-Żydecka et al. (178) analyzed the changes in the GM composition and body weight gain in individuals treated with SGAs. The findings showed increased weight gain in both experimental rodents and humans, as well as alterations in the GM, with primarily a higher abundance of *Firmicutes* than *Bacteroidetes*. In addition, treatment with SGAs (olanzapine and risperidone) was associated with metabolic changes and inflammation in experimental animals. Similarly, Davey et al. (179) reported altered microbial composition linked to chronic olanzapine treatment in a rat model, as well as increased plasma levels of IL-8 and IL-1β in female rats only. According to Kao et al. (180), adding the prebiotic B-GOS^®^ to olanzapine treatment may prevent weight gain and improve cognitive performance in psychosis. Furthermore, in a rat model, B-GOS^®^ reduced the abundance of several bacteria in the *Firmicutes* phylum and increased the abundance of *Bifidobacterium* spp. but olanzapine treatment, either alone or in combination with B-GOS^®^, had no effect. Although clinical improvements and increases in BMI were noted in patients with schizophrenia treated with olanzapine, no changes in GM composition were observed (181). In children and adolescents, however, chronic risperidone treatment decreased the *Bacteroidetes: Firmicutes* ratio and elevated BMI (182). *Ex vivo*, aripiprazole reduced the abundance of *Lachnospiraceae*, *Lactobacillaceae*, and *Erysipelotrichaceae*, which was accompanied by lower levels of SCFAs. Co-administration of *Lactobacillus rhamnosus* and *Bifidobacterium longum* with aripiprazole restored SCFA levels (183). However, there is no direct evidence that alterations in GM could mediate changes in bone structure with AP use.

## 8 Osteoporosis induced by antidepressant drugs and the gut microbiota

Antidepressants (ADs) are a class of medications used to treat depression and other mental health issues, including obsessive-compulsive disorder, anxiety, and post-traumatic stress disorder. They most likely exert their effects by increasing extracellular levels of monoamine neurotransmitters (e.g., serotonin, norepinephrine, dopamine), or by stimulating synaptic plasticity and neurogenesis (184, 185). Key classes include selective serotonin reuptake inhibitors (SSRIs, e.g., citalopram, escitalopram, fluoxetine, fluvoxamine, paroxetine), serotonin norepinephrine reuptake inhibitors (SNRIs, e.g., venlafaxine, duloxetine), tricyclic ADs (TCAs, e.g., amitriptyline, doxepin, imipramine), and miscellaneous ADs, with SSRIs being the most common type (186, 187). Multiple studies examined the association between depression and bone health, specifically BMD and fracture risk, with varying findings due to different methodologies and diagnostic criteria. While some studies found no significant correlations, others confirmed detrimental effects of depression on bone health (188, 189). Although the exact mechanisms are unclear, it is generally accepted that depression adversely influences bone health (190). Its pharmacological treatment with ADs is one of the possible causes, and it can result in the development of osteoporosis.

The mechanisms by which depression negatively influence bone health include 5-HT-mediated suppression of osteoblast function and disturbed hormonal balance (mainly increasing cortisol). Elevated cortisol levels have been found to inhibit osteoblast expression of IGF-1, leading to decreased BMD (191). In addition, high cortisol concentrations may directly affect the RANK/RANKL/OPG system by increasing RANKL expression, thereby promoting osteoclast differentiation and activity (192). Since osteoblasts, osteoclasts, and osteocytes generally express 5-HT receptors, modifying 5-HT levels with ADs can have a harmful impact on bone mass and metabolism (193). Another proposed mechanism involves a direct effect of certain ADs (mainly SSRIs) on bone cells (osteoclasts, osteoblasts), leading to inhibited bone formation, elevated bone resorption, or both (194).

According to a recent cross-sectional study by Rajha et al. (187), AD therapy was associated with an increased risk of osteoporosis (by 44%). In addition, concomitant AD use worsened osteoporosis in adult women. In this regard, a current meta-analysis by Mercurio et al. (195) found that use of ADs, particularly SSRIs, was associated with reduced BMD. In general, long-term SSRI treatment was linked to decreased vertebral and non-vertebral BMD, increased risk of clinical fractures, and an elevated likelihood of falls in other studies (196, 197). According to Doğan Bulut et al. (198), SSRIs may put postmenopausal women at a higher risk of developing low BMD in the femoral and lumbar regions compared to SNRIs. Agarwal et al. (199) reported that current AD therapy is associated with cortical bone deficits and reduced physical function in elderly women, but the effects may be class-specific. When assessed by AD class, reduced BMD was identified only in SSRI users at the radius and only in SNRI users at the proximal tibia. A higher risk of non-vertebral fractures was documented in older individuals treated with TCAs, according to Ziere et al. (196). In contrast, other research has shown that TCA treatment was not linked to raising rate of bone loss at the total hip (200).

Growing evidence indicates that SSRIs influence the composition, diversity, and richness of microbial communities within the gut (23, 201). Although the mechanisms linking SSRIs, the GM, and bone health remain unclear, these drugs may promote osteoporosis by disrupting microbiota-derived metabolites and immune signaling pathways (23). In human twins, SSRI treatment was negatively associated with the abundance of the family *Turicibacteraceae* (202). Citalopram altered the abundance of *Ruminococcaceae*, *Desulfovibrionaceae*, and *Lactobacillaceae* in mice exposed to chronic restraint stress, which induced depression- and anxiety-like behaviors (203). In a murine model, escitalopram increased α-diversity of GM, with differences in GM observed between responders and non-responders. *Ruminococcaceae* and *Lactobacillaceae* were notably absent in non-responding mice. Moreover, fifteen serum metabolites mainly involved in phospholipid metabolism discriminated responders from non-responders (204). Lukic et al. (205) evaluated the effects of multiple ADs, including fluoxetine, escitalopram, venlafaxine, duloxetine, and desipramine, on the GM composition using a mice model. ADs reduced bacterial richness and increased β-diversity of gut bacteria. At the genus level, decreased abundance of *Ruminococcus* and *Adlercreutzia* was observed in AD-treated mice. Furthermore, supplementation with *Ruminococcus flavefaciens* attenuated the antidepressant effect of duloxetine, whereas supplementation with *Adlercreutzia equolifaciens* had no effect. AD use is associated with lower BMD, highlighting the importance of identifying novel therapies that preserve bone health in patients with psychiatric disorders. A recent study by Wan et al. (206) investigated whether the GM modulates positive effects of arketamine (a novel AD) on decreased BMD in mice subjected to chronic social defeat stress (CSDS). Arketamine treatment attenuated anhedonia-like behavior and improved reduced cortical (and total) BMD in the femoral neck. In addition, correlations have been found between the abundance of certain microbiota (and six plasma metabolites) and BMD. Compared with the CSDS + saline group, the abundance of *Acetanaerobacterium elongatum*, *Blautia faecis*, and *Mucispirillum schaedleri* was lower in the CSDS + arketamine group, while the abundance of *Parabacteroides merdae* was significantly higher.

The information presented so far suggests that different drugs can cause secondary osteoporosis through multiple mechanisms. Several of the drugs listed above share similar mechanisms of their negative impact on bone health. These mechanisms are summarized in **Table 1**, including those linked to the development of osteoporosis and alterations in the GM. A current pharmacovigilance analysis using the FDA Adverse Event Reporting System database by Wang et al. (207) revealed that the highest incidence of osteoporosis-related side effects was observed with ARs, particularly tenofovir disoproxil and its combination therapies. Although GCs had a lower proportion of reports, their rapid onset of action emphasizes the need for vigilance. These results highlight the need to monitor bone health in long-term drug users and offer practical evidence for drug safety management. Liu et al. (208) pointed out important differences in the risk of drug-induced osteoporosis between the sexes and emphasized the need for targeted pharmacovigilance strategies. According to their findings, 68 drugs were associated with drug-induced osteoporosis, including 15 drugs with a potential risk specific to men and 26 drugs with a potential risk specific to women. However, some drugs, such as tenofovir disoproxil and esomeprazole, were associated with both sexes. Drugs such as upadacitinib (an oral Janus kinase 1-selective inhibitor and a disease-modifying antirheumatic drug) showed patterns of early-onset failure, while others, such as tenofovir, showed cumulative patterns of risk with long-term use.

**Table 1:** Effects of selected drugs on bone health linked to the development of secondary osteoporosis and changes in the gut microbiota.

Drugs	Osteoporosis	Dynamics of gut microbiota at the genus and family level
Glucocorticoids (GCs)	↓ Wnt pathway ↑ RANKL/OPG pathway ↑ M-CSF ↑ apoptosis of osteoblasts and osteocytes ↓ estrogen, Ca ↓ IGF-1 and growth hormone ↑ PTH	↑ *Desulfovibrionaceae* ↓ *Erysipelotrichaceae* ↓ *Burkholderiaceae* ↑ *Anaerobacterium* ↓ *Eisenbergiella* ↓ *Alistipes* ↓ *Clostridium XIVb* ↓ *Lactobacillus* ↑ *Bifidobacterium* ↑ *Lactobacillus* ↓ *Mucispirillum*
Aromatase inhibitors (AIs)	↓ estrogen ↑ IL–1, TNF-α ↑ differentiation of osteoclasts ↓ differentiation of osteoblasts ↑ PYD, DPD ↓ 25-hydroxyvitamin D	↑ *Oscillospiraceae* ↑ *Veillonella* ↓ *Coprococcus* ↓ *Ruminococcus* ↓ *Corynebacterium* ↓ *Butyricicoccus* ↓ *Holdemania* ↓ *Haemophilus*
Proton pump inhibitors (PPIs)	↓ Ca, Mg ↓ vitamin B12 ↑ HCY ↑ PTH ↓ the vacuolar-type H^+^-ATPase	↓ *Ruminococcaceae* ↓ *Lachnospiraceae* ↑ *Enterococcus* ↑ *Streptococcus* ↑ *Staphylococcus* ↑ *Rothia*
Antiretrovirals (ARs)	↓ vitamin D ↓ DNA polymerase-γ ↑ leptin ↓ calcium hydroxyapatite chronic acidosis ↑ NF-κB, RANKL ↓ α1-hydroxylase ↓ Wnt pathway	↑ *unclassified Lachnospiraceae* ↑ *Dorea* ↑ *Megasphaera* ↑ *Mitsuokella* ↑ *Ruminococcus*
Antiepileptics (AEDs)	activation of PXR ↓ vitamin D, Ca ↑ PTH, HCY ↓ estrogen ↑ IL-1, IL-6, TNF-α ↑ RANKL ↓ differentiation of osteoblasts ↑ differentiation of osteoclasts	↑ *Arthrobacter* ↑ *Achromobacter* ↑ *Gordonia* ↑ *Shinella*
Antipsychotics (APs)	↑ prolactin ↓ vitamin D ↓ gonadotropins potential interactions with dopaminergic, serotonergic, and adrenergic receptors in bone cells ↓ physical activity	↑ *Erysipelotrichaceae* ↑ *Butyricimonas* ↑ *Blautia* ↑ *Paraprevotella*
Antidepressants (ADs)	↑ serotonin ↑ cortisol ↑ differentiation of osteoclasts ↓ differentiation of osteoblasts	↓ *Turicibacteraceae* ↓ *Ruminococcus* ↓ *Adlercreutzia*

decrease of value, ↓; increase of value, ↑; calcium, Ca; deoxypyridinoline, DPD; glucocorticoid, GC; homocysteine, HCY; insulin-like growth factor-1, IGF-1; interleukin, IL; macrophage colony stimulating factor, M-CSF; osteoprotegerin, OPG; parathyroid hormone, PTH; pregnane X receptor, PXR; pyridinoline, PYD; receptor activator of NF-κB ligand, RANKL; tumor necrosis factor-alpha, TNF-α.

## 9 Therapy of drug-induced osteoporosis

Therapy for drug-induced osteoporosis depends on the specific drug, but generally involves a combination of medications, lifestyle changes, and preventive measures. Only a few anti-osteoporotic drugs have official indications for osteoporosis induced by certain drug classes, namely GCs and AIs. Osteoporotic changes observed in patients treated with other drug classes described in this review are generally considered to be common in the general population. However, in addition to official recommendations, there are reports of the use of various medications to treat drug-induced osteoporosis.

Bisphosphonates, which are antiresorptive drugs that inhibit osteoclast activity and include alendronate, risendronate, and zoledronic acid, are often used as first-line therapy for secondary osteoporosis caused by GCs and AIs (209). They are also used for treatment of ART-induced osteoporosis (132, 210), and AED-induced osteoporosis (211, 212). Denosumab, an antiresorptive drug, is also used to treat GC-induced osteoporosis, AI-induced osteoporosis, as well as osteoporosis caused by ARTs and ADs (209, 210, 213, 214). The use of teriparatide, an anabolic agent, is clinically limited to 24 months (215) and is therefore used as first aid for the sharp decline in BMD caused by GCs and ARTs (85, 216–218). Adequate intake of Ca and vitamin D, weight-bearing exercise, and BMD monitoring are also recommended as important parts of prevention and treatment. Probiotic and prebiotic supplementation could represent a future option if supported by convincing clinical evidence.

## 10 Conclusions

Many commonly administered pharmacological drugs, such as GCs, AIs, PPIs, ARs, AEDs, APs, and ADs, can negatively affect bone homeostasis through reduced BMD, increased fracture risk, and manifest as secondary osteoporosis. Furthermore, the bidirectional relationship between these drugs and the GM, where the GM can also influence drug metabolism and efficacy, adds another layer of complexity to these interactions.

There are several ways in which different drugs can cause secondary osteoporosis. Many medications have similar mechanisms of detrimental impact on bone health, including the development of osteoporosis (e.g., they reduce levels of estrogen, Ca, vitamin D, increase PTH levels, support osteoclastogenesis, inhibit osteoblastogenesis) and alterations in GM. Since GM plays a key role in regulating bone remodeling by modulating osteoclast-driven resorption and osteoblast-driven formation, drug-induced changes in microbial composition, diversity, and metabolic activity may influence skeletal integrity and systemic physiology. GCs alter multiple bacterial taxa, including reductions in *Erysipelotrichaceae* and *Burkholderiaceae* and increases in *Desulfovibrionaceae*. At the same time, genera such as *Anaerobacterium* and *Bifidobacterium* are elevated, and *Alistipes, Eisenbergiella, Clostridium XIVb*, and *Mucispirillum* are decreased. The commensal genus *Lactobacillus*, belonging to the phylum Firmicutes, shows variable responses depending on the GC type: dexamethasone increases its abundance, potentially contributing to anti-inflammatory effects, whereas methylprednisolone decreases it. Similarly, other drug classes impact GM: AEDs increase *Arthrobacter, Achromobacter, Gordonia*, and *Shinella* in animal models; AIs in postmenopausal breast cancer patients modify taxa such as *Oscillospiraceae, Veillonella*, and reduce *Coprococcus, Ruminococcus*, and others; PPIs decrease microbial diversity, deplete beneficial SCFA-producing bacteria (*Ruminococcaceae, Lachnospiraceae*), and enrich potentially pathogenic genera (*Enterococcus, Streptococcus, Staphylococcus, Rothia*). ART in HIV-infected individuals is associated with *elevated Lachnospiraceae, Dorea, Megasphaera, Ruminococcus*, and *Mitsuokella* in women with low BMD. APs and ADs also modulate gut microbial composition, with typical APs increasing *Erysipelotrichaceae, Blautia*, and *Paraprevotella*, while SSRIs and other ADs reduce *Turicibacteraceae*, butyrate-producers such as *Ruminococcus*, and anti-inflammatory *Adlercreutzia*. Collectively, these observations highlight that diverse pharmacological drugs can significantly reshape gut microbial communities, often in a drug- and context-specific manner, underscoring the complex interplay between the GM and host physiology.

It is currently largely unresolved how certain drugs, GM, and bone health are related. There is little or no direct evidence that drug-induced GM alterations influence changes in bone turnover, BMD, or fracture risk for most of the drug classes described (e.g., AIs, AEDs, APs, ADs). The evidence is largely preclinical with little translational or clinical validation, even for GIOP, which has been more thoroughly investigated. The data now available for PPIs even suggest that changes in GM may not mediate the observed effects on bone structural parameters.

It was found that the highest incidence of osteoporosis-related side effects was reported with ARs, particularly with tenofovir disoproxil and its combination therapies. Despite the smaller number of reports, the rapid response of GC’s effect on bone health highlights the importance of vigilance. Targeted pharmacovigilance methods must also take into account sex differences in the risk of drug-induced osteoporosis. However, effects of some drugs, such as tenofovir disoproxil and esomeprazole, are associated with both sexes. In general, pharmacological therapy, lifestyle changes, adequate intake of Ca and vitamin D, weight-bearing exercise, and preventive BMD monitoring are some of the therapeutic approaches that have shown promise in the treatment of drug-induced osteoporosis. Probiotic and prebiotic supplementation may also be useful in treating this condition if supported by robust scientific evidence. However, there are still a small number of preclinical and clinical studies investigating the relationship between drug-induced osteoporosis and GM. Observational and interventional human studies in this area are crucial to definitively demonstrate this relationship. Therefore, there are still many unanswered questions in the field of osteomicrobiology that need to be answered.
